# Cost-effectiveness of nivolumab plus ipilimumab versus lenvatinib or sorafenib for unresectable hepatocellular carcinoma in China: a modeling study with price threshold analysis

**DOI:** 10.3389/fphar.2026.1860312

**Published:** 2026-06-30

**Authors:** Rui Feng, Liman Huo, Yun-Feng Diao, Yajing Wang, Mei Zhou, Ying Zheng, Qi Lv

**Affiliations:** 1 School of Disaster and Emergency Medicine, Tianjin University, Tianjin, China; 2 Department of Pharmacy, The Fourth Hospital of Hebei Medical University, Shijiazhuang, China; 3 Institute of Traumatic Brain Injury and Neurology, Characteristic Medical Center of PAPF, Tianjin, China

**Keywords:** cost-effectiveness analysis, economic evaluation, hepatocellular carcinoma, immune checkpoint inhibitors, partitioned survival model, threshold analysis

## Abstract

**Background:**

Nivolumab plus ipilimumab has demonstrated survival benefits as first-line therapy for unresectable hepatocellular carcinoma (HCC). However, the high treatment cost may limit its economic value in China. This study evaluated the cost-effectiveness of nivolumab plus ipilimumab versus lenvatinib or sorafenib for unresectable HCC from the perspective of the Chinese healthcare system.

**Methods:**

A partitioned survival model (PSM) with progression-free survival (PFS), progressed disease (PD), and death health states was developed over a 10-year time horizon. Clinical data were obtained from the phase III CheckMate 9DW trial. Overall survival (OS) and PFS data were reconstructed from published Kaplan–Meier curves and extrapolated using alternative parametric and flexible survival models. Model selection was based on statistical fit, visual inspection, and clinical plausibility. Structural uncertainty was explored through 625 survival model combinations and multiple scenario analyses involving time horizons, post-progression treatment, treatment exposure, adverse event (AE) management, utility values, and non-drug medical costs. Threshold and price–willingness-to-pay (WTP) analyses were conducted to evaluate the impact of treatment price reductions on cost-effectiveness outcomes.

**Results:**

In the base-case analysis, nivolumab plus ipilimumab increased total costs (USD 162,437.52 vs. USD 81,990.70) and quality-adjusted life years (QALYs) (1.89 vs. 1.40), resulting in an incremental cost-effectiveness ratio (ICER) of USD 164,177.18/QALY, substantially exceeding the Chinese WTP threshold of USD 28,972.38/QALY. Across all 625 survival model combinations and scenario analyses, ICERs consistently remained above the predefined WTP threshold. Scenario analyses demonstrated that the economic conclusions were generally robust to alternative assumptions regarding survival extrapolation, utility inputs, downstream treatment, treatment exposure, and healthcare costs. Price-threshold analyses indicated that an approximate 67% reduction in treatment cost would be required for nivolumab plus ipilimumab to reach the conventional Chinese WTP threshold.

**Conclusion:**

At current prices, nivolumab plus ipilimumab is unlikely to be cost-effective as a first-line treatment for unresectable HCC in China. This conclusion remained consistent across extensive structural and parameter uncertainty analyses. Substantial price reductions may be required to improve the economic value of dual immunotherapy in the Chinese healthcare setting.

## 1 Introduction

Hepatocellular carcinoma (HCC) remains a major cause of cancer-related mortality worldwide, particularly in Asia. In China, a substantial proportion of patients are diagnosed at an unresectable stage, for which systemic therapy is the main treatment option (Llovet et al., 2021). In recent years, immune checkpoint inhibitors have reshaped the treatment landscape of unresectable HCC and demonstrated clinically meaningful survival benefits in the first-line setting (Reig et al., 2022; Sangro et al., 2021).

Among currently available immunotherapy-based strategies, nivolumab plus ipilimumab demonstrated superior overall survival (OS) compared with tyrosine kinase inhibitors (TKIs) in previously untreated patients with unresectable HCC in the phase III CheckMate 9DW trial (Yau et al., 2025). Importantly, this survival benefit was observed in a disease setting traditionally considered less responsive to immunotherapy, highlighting the potential clinical value of dual immune checkpoint blockade in advanced HCC. Based on these findings, the regimen has been incorporated into the National Comprehensive Cancer Network (NCCN) Clinical Practice Guidelines in Oncology for HCC (Version 1.2025) as a category 2A recommended first-line treatment option (National Comprehensive Cancer Network). However, the high acquisition cost of dual immunotherapy may limit its affordability and economic value, particularly in healthcare systems with constrained resources.

Economic evaluation has therefore become increasingly important for informing treatment selection and healthcare resource allocation. Several studies have evaluated the cost-effectiveness of nivolumab plus ipilimumab for unresectable HCC (Wu et al., 2026; Luo et al., 2026). However, long-term economic outcomes in oncology are highly dependent on survival extrapolation beyond the observed trial period, and different modeling assumptions may substantially influence estimated costs and quality-adjusted life years (QALYs) (Latimer, 2013). The impact of structural uncertainty related to survival extrapolation and downstream treatment assumptions therefore warrants further evaluation.

Despite the growing use of immunotherapy in advanced HCC, evidence regarding the economic value of nivolumab plus ipilimumab in China remains limited. Therefore, this study evaluated the cost-effectiveness of nivolumab plus ipilimumab compared with lenvatinib or sorafenib from the perspective of the Chinese healthcare system. In addition to the base-case analysis, extensive scenario and sensitivity analyses were conducted to assess the robustness of the results under alternative modeling assumptions and parameter inputs.

## 2 Materials and methods

### 2.1 Study population

This analysis was based on the population enrolled in the phase III randomized controlled CheckMate 9DW trial (Yau et al., 2025). The target population included adults aged ≥18 years with histologically or radiologically confirmed unresectable HCC who had not received prior systemic therapy.

Eligible patients were required to have at least one measurable lesion according to the Response Evaluation Criteria in Solid Tumours (RECIST) version 1.1, an Eastern Cooperative Oncology Group (ECOG) performance status of 0–1, and a Child–Pugh score of 5–6. These eligibility criteria reflected a trial population with preserved liver function and good performance status; therefore, the generalizability of the findings to routine clinical practice may be limited.

### 2.2 Treatment strategies

Two first-line treatment strategies were evaluated. In the experimental arm, nivolumab (1 mg/kg) plus ipilimumab (3 mg/kg) was administered every 3 weeks for four induction cycles, followed by nivolumab maintenance therapy (480 mg every 4 weeks) until disease progression, unacceptable toxicity, or a maximum treatment duration of 2 years.

In the comparator arm, treatment consisted of TKIs, including lenvatinib or sorafenib, at the investigator’s discretion. Lenvatinib was administered at 8 mg once daily for patients weighing <60 kg and 12 mg once daily for those weighing ≥60 kg, whereas sorafenib was administered at 400 mg twice daily. Based on treatment distributions reported in the CheckMate 9DW trial, lenvatinib and sorafenib accounted for 85% and 15% of the comparator group, respectively, and a weighted treatment cost was incorporated into the model. Because body weight distributions were not reported in the original trial, the proportion of patients weighing ≥60 kg was derived from the phase III REFLECT trial (69%) and was used to estimate weighted lenvatinib dosing (Kudo et al., 2018).

After disease progression, a proportion of patients received subsequent systemic therapy. According to the CheckMate 9DW trial, post-progression treatment was administered to 38% of patients in the experimental arm and 52% of patients in the comparator arm. In the model, atezolizumab plus bevacizumab was selected as representative post-progression therapy. Detailed treatment schedules and cost assumptions are provided in Supplementary Table S3. Patients who did not receive subsequent systemic therapy were assumed to receive best supportive care (BSC), and the corresponding costs were incorporated as a one-time cost upon progression.

### 2.3 Model structure

**FIGURE 1:**
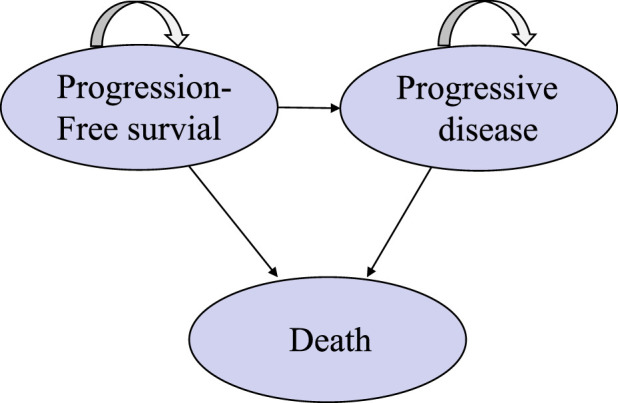
Structure of the PSM Abbreviations: PD, progressed disease; PFS, progression-free survival; PSM, partitioned survival model.

A partitioned survival model (PSM) was developed in R software (version 4.4.2) to evaluate the cost-effectiveness of the treatment strategies. The model comprised three mutually exclusive health states: progression-free survival (PFS), progressed disease (PD), and death (Figure 1).

Health-state occupancy over time was estimated using reconstructed and extrapolated OS and PFS data from the CheckMate 9DW trial. The proportion of patients remaining in the PFS state was derived directly from the PFS curve, whereas the proportion in the PD state was estimated as the difference between the OS and PFS curves. Parametric and flexible survival models were fitted to the reconstructed survival data. Model selection was based on statistical goodness-of-fit criteria, including the Akaike information criterion (AIC) and Bayesian information criterion (BIC), together with visual inspection and clinical plausibility. The original Kaplan–Meier curves and reconstructed survival data are presented in Supplementary Figure S1.

For the base-case analysis, the RP-odds model and Weibull distribution were selected for PFS and OS in the control group, respectively, whereas the RP-hazard model and gamma distribution were selected for the experimental group. A 21-day cycle length was adopted to reflect treatment administration schedules in the CheckMate 9DW trial. A 10-year time horizon was adopted to approximate a lifetime horizon for patients with unresectable HCC. In accordance with the China Guidelines for Pharmacoeconomic Evaluations (2025), both costs and health outcomes were discounted at an annual rate of 4.5% (Wu and Liu, 2025).

### 2.4 Survival data processing and extrapolation

A broad range of survival models was evaluated to identify appropriate distributions for long-term extrapolation. Candidate models included seven standard parametric distributions (Exponential, Weibull, Gompertz, Log-normal, Log-logistic, Generalized gamma, and Gamma) and five flexible modeling approaches, including fractional polynomials (FP), restricted cubic splines (RCS), Royston–Parmar (RP) models, generalized additive models (GAM), and mixture cure models (MCM).

Model selection was initially based on statistical goodness-of-fit criteria, including the Akaike information criterion (AIC) and Bayesian information criterion (BIC). Models with poor statistical fit or implausible extrapolation patterns were excluded. The remaining candidate models were further assessed for clinical plausibility, including consistency with the natural history of advanced HCC, absence of implausible curve crossings (e.g., PFS exceeding OS), and plausibility of the corresponding hazard functions over time. Detailed model-fitting results are presented in Supplementary Table S1.

Visual inspection was additionally performed by comparing fitted curves with reconstructed Kaplan–Meier data to assess agreement during the observed follow-up period and the plausibility of long-term extrapolation. Fitted and extrapolated survival curves are presented in Supplementary Figures S2, S3. Final model selection was based on an integrated assessment of statistical fit, visual inspection, and clinical plausibility.

To evaluate structural uncertainty associated with survival extrapolation, multiple clinically plausible candidate models were retained for scenario analyses. The base-case analysis used selected OS and PFS models for each treatment group according to their overall performance across statistical fit, visual inspection, and clinical plausibility. Alternative model specifications were incorporated into scenario analyses to assess the impact of different survival assumptions on cost-effectiveness outcomes.

In the base-case analysis, the RP-hazard and RP-odds models were selected for PFS in the experimental and control groups, respectively, whereas the Gamma and Weibull distributions were selected for OS in the experimental and control groups, respectively. The resulting OS and PFS functions were subsequently used to estimate health-state occupancy over time within the PSM. Long-term extrapolated survival estimates were additionally assessed against expected clinical trajectories in advanced HCC.

### 2.5 Cost and utility inputs

The analysis was conducted from the perspective of the Chinese healthcare system and included only direct medical costs. Cost categories included drug acquisition costs for nivolumab plus ipilimumab and the comparator treatments (lenvatinib and sorafenib), disease management costs, post-progression treatment costs, BSC costs, and costs associated with adverse event (AE) management (Z et al., 2025; Lin et al., 2024). Drug acquisition costs were estimated using national bid-winning and negotiated price data obtained from the Wuxu Data Platform in China (https://www.wuxuwang.com/).

Disease management costs included laboratory testing and imaging examinations. Costs related to disease management, BSC, AE management, and health-state utility values were derived from published literature (Qin et al., 2018; Leng et al., 2022; National Healthcare Security Administration NHSA, 2025; Zheng et al., 2024; Nafees et al., 2008; Nafees et al., 2017; National Institute for Health and Care Excellence NICE, 2015). To simplify the model, only grade ≥3 AEs with an incidence of ≥2% in either treatment group were included, and all AE-related costs and disutilities were assumed to occur during the first treatment cycle. AE incidence data were obtained from the CheckMate 9DW trial.

Drug dosing was estimated using average height and body weight data for the Chinese adult population reported in the Report on Nutrition and Chronic Diseases in China (2020) (Chinese Nutrition Society, 2020), weighted according to the sex distribution observed in the CheckMate 9DW trial. Mean body weight was assumed to be 67.7 kg, and mean body surface area was estimated at 1.77 m^2^ using the Du Bois formula.

Health-state utility values were derived from published studies in patients with HCC. Utility values for the PFS and PD states were 0.76 and 0.68, respectively (Zheng et al., 2024). The willingness-to-pay (WTP) threshold was set at two times the *per capita* gross domestic product (GDP) of China in 2025, corresponding to USD 28,972.38 per QALY. Key model parameters are summarized in Table 1.

**TABLE 1:** Model parameters used in the economic evaluation.

Parameter	Base value	Lower bound	Upper bound	Distribution	Source
Induction treatment cost in experimental group (USD per cycle)	11,537.53	8,653.15	14,421.91	Gamma	Wuxu Data Platform
Maintenance treatment cost in experimental group (USD per cycle)	4,840.11	3,630.08	6,050.14	Gamma	Wuxu Data Platform
Treatment cost in control group (USD per cycle)	414.20	310.65	517.75	Gamma	Wuxu Data Platform
Laboratory testing cost (USD per cycle)	38.10	28.58	47.63	Gamma	National Healthcare Security Administration NHSA (2025)
Imaging cost (USD per cycle)	38.66	29.00	48.33	Gamma	National Healthcare Security Administration NHSA (2025)
Post-progression treatment cost in experimental group (USD per cycle)	2,371.34	1,778.51	2,964.18	Gamma	Wuxu Data Platform
Post-progression treatment cost in control group (USD per cycle)	3,244.99	2,433.74	4,056.24	Gamma	Wuxu Data Platform
BSC cost in experimental group (USD per phase)	7,934.75	5,951.06	9,918.44	Gamma	Qin et al. (2018)
BSC cost in control group (USD per phase)	6,143.12	4,607.34	7,678.90	Gamma	Qin et al. (2018)
End-of-life care cost (USD per terminal phase)	13,572.00	10,179.00	16,965.00	Gamma	Leng et al. (2022)
Utility of PFS	0.76	0.57	0.95	Beta	Zheng et al. (2024)
Utility of PD	0.68	0.51	0.85	Beta	Zheng et al. (2024)
Annual discount rate	0.045	0	0.05	Beta	Wu and Liu (2025)
Cost of managing AEs in experimental group (USD per event)	317.88	238.41	397.35	Gamma	(Lin et al., 2024; Qin et al., 2018)
Cost of managing AEs in control group (USD per event)	372.21	279.16	465.26	Gamma	(Lin et al., 2024; Qin et al., 2018)
Disutility associated with AES in experimental group	0.02	0.015	0.03	Beta	(Nafees et al., 2008; Nafees et al., 2017; National Institute for Health and Care Excellence NICE, 2015)
Disutility associated with AEs in control group	0.04	0.03	0.05	Beta	(Nafees et al., 2008; Nafees et al., 2017; National Institute for Health and Care Excellence NICE, 2015)

Abbreviations: AE, adverse event; AEs, adverse events; BSC, best supportive care.

### 2.6 Sensitivity analyses

To evaluate parameter uncertainty, both one-way sensitivity analysis and probabilistic sensitivity analysis (PSA) were performed. In the one-way sensitivity analysis, key model parameters were varied within predefined ranges derived from published literature or plausible assumptions. When confidence intervals were unavailable, ranges of ±25% of base-case values were applied. The influence of individual parameters on the incremental cost-effectiveness ratio (ICER) was evaluated using tornado diagrams.

PSA was conducted using Monte Carlo simulation with probability distributions assigned according to parameter type. Gamma distributions were applied to cost parameters because costs were non-negative and right-skewed, whereas Beta distributions were used for utility values and probabilities because these parameters were bounded between 0 and 1. A total of 5,000 simulation iterations were performed to characterize overall model uncertainty. Results were presented as cost-effectiveness scatter plots and cost-effectiveness acceptability curves (CEACs).

### 2.7 Scenario analyses

#### 2.7.1 Survival model uncertainty

To evaluate structural uncertainty related to survival extrapolation, scenario analyses were conducted using alternative survival model specifications. The five most clinically plausible and statistically well-fitting models for OS and PFS in each treatment group were retained, generating 625 survival model combinations. ICERs were recalculated for each combination to assess the impact of alternative survival assumptions on cost-effectiveness outcomes.

Additional scenario analyses were performed using alternative time horizons of 5 and 15 years alongside the base-case 10-year horizon. The 5-year horizon was used to reduce reliance on long-term extrapolation beyond the observed trial period, whereas the 15-year horizon was used to explore the potential impact of more prolonged survival benefits associated with immunotherapy. ICERs were recalculated under each time-horizon scenario to evaluate the sensitivity of the economic results to extrapolated survival duration.

#### 2.7.2 Utility value uncertainty

To evaluate uncertainty associated with health-state utility values, scenario analyses were conducted using alternative utility estimates derived from published literature. A targeted literature search was performed in PubMed to identify studies reporting utility values in patients with HCC. Relevant health technology assessment reports and published economic evaluations were also reviewed.

Studies reporting utility estimates for the PFS and PD health states in patients with HCC were reviewed. Preference-based utility measures derived from EQ-5D instruments were prioritized when available. Alternative utility estimates from the literature were incorporated into scenario analyses to assess the impact of utility uncertainty on cost-effectiveness outcomes.

#### 2.7.3 Treatment and healthcare cost uncertainty

Post-progression treatment strategies in the CheckMate 9DW trial were heterogeneous and included both systemic and non-systemic therapies. Therefore, additional scenario analyses were conducted to evaluate uncertainty related to downstream treatment assumptions, treatment exposure, toxicity burden, and healthcare resource utilization.

In the base-case analysis, patients receiving subsequent systemic therapy were assumed to receive atezolizumab plus bevacizumab as representative post-progression treatment. To assess the impact of uncertainty in downstream treatment and healthcare resource utilization, the following alternative scenarios were evaluated:

1. BSC only after disease progression;
2. post-progression treatment costs reduced by 50%; and
3. post-progression treatment costs increased by 50%.

Additional scenario analyses were performed by increasing AE management costs to 1.5-fold and 2-fold of base-case values to explore the potential impact of greater toxicity-related healthcare utilization in routine clinical practice.

Because treatment exposure and relative dose intensity in routine practice may differ from those observed in clinical trials, an additional scenario analysis was conducted in which experimental treatment costs were reduced to 80% of base-case values. This scenario was intended to reflect potential treatment interruption, dose reduction, or lower treatment exposure in real-world settings.

To assess potential regional variation in healthcare utilization and medical pricing across China, non-drug medical costs, including disease management and supportive care costs, were varied by ±25% in additional scenario analyses.

These scenario analyses were conducted to evaluate the robustness of the economic results to alternative assumptions regarding downstream treatment, toxicity burden, treatment exposure, and healthcare resource utilization.

### 2.8 Price threshold and decision-space analysis

In the price-threshold analysis, first-line treatment costs in the experimental arm were progressively reduced while all other model parameters remained unchanged. ICERs were recalculated at each price level to estimate the magnitude of price reduction required for nivolumab plus ipilimumab to reach commonly used Chinese WTP thresholds.

Additional price–WTP scenario analyses were conducted across different treatment prices and WTP thresholds corresponding to 1×, 2×, and 3× GDP *per capita* in China. Heatmaps were generated to illustrate how variations in treatment price and WTP thresholds influenced the cost-effectiveness results.

In addition, the reporting of this economic evaluation was conducted in accordance with the Consolidated Health Economic Evaluation Reporting Standards (CHEERS) 2022 checklist, as summarized in Supplementary Table S4.

## 3 Results

### 3.1 Base-case analysis

The base-case analysis (Table 2) showed that nivolumab plus ipilimumab was associated with higher costs and greater QALYs than the comparator strategy, resulting in an ICER of USD 164,177.18/QALY. This value substantially exceeded the WTP threshold of twice the *per capita* GDP of China in 2025 (USD 28,972.38/QALY), indicating that nivolumab plus ipilimumab was unlikely to be cost-effective under base-case conditions.

**TABLE 2:** Base-case cost-effectiveness results.

Group	Total cost (USD)	Total QALYs	Incremental cost (USD)	Incremental QALYs	ICER (USD/QALY)
Experimental group	162,437.52	1.89	80,446.82	0.49	164,177.18
Control group	81,990.70	1.40	–	–	–

Abbreviations: ICER, incremental cost-effectiveness ratio; QALY, quality-adjusted life year.

### 3.2 Sensitivity analyses

**FIGURE 2:**
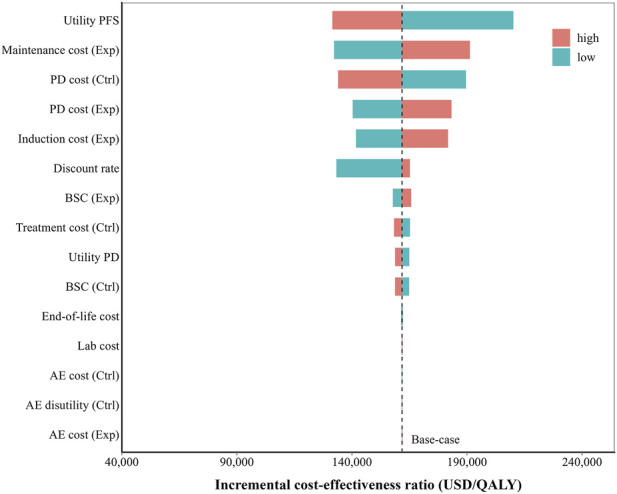
Tornado diagram of one-way sensitivity analysis.

The one-way sensitivity analysis results (Figure 2) showed that the utility value of the PFS state had the greatest influence on the ICER, followed by maintenance-phase treatment costs in the experimental group. Costs associated with the PD state in both treatment arms and induction-phase treatment costs in the experimental group also influenced the model results, whereas the discount rate had a comparatively smaller impact on the ICER.

**FIGURE 3:**
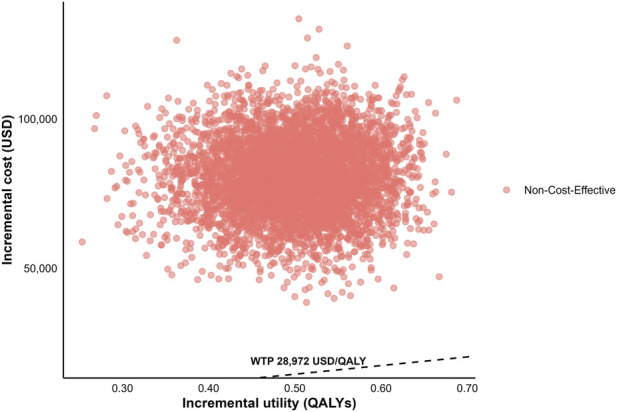
Cost-effectiveness scatter plot from probabilistic sensitivity analysis.

The PSA results (Figure 3) showed that most of the 5,000 Monte Carlo simulations were distributed in the northeast quadrant of the cost-effectiveness plane, indicating higher costs and greater effectiveness for nivolumab plus ipilimumab compared with the comparator strategy. Most simulated ICERs exceeded the WTP threshold of USD 28,972.38/QALY, suggesting that nivolumab plus ipilimumab was unlikely to be cost-effective at current prices.

**FIGURE 4:**
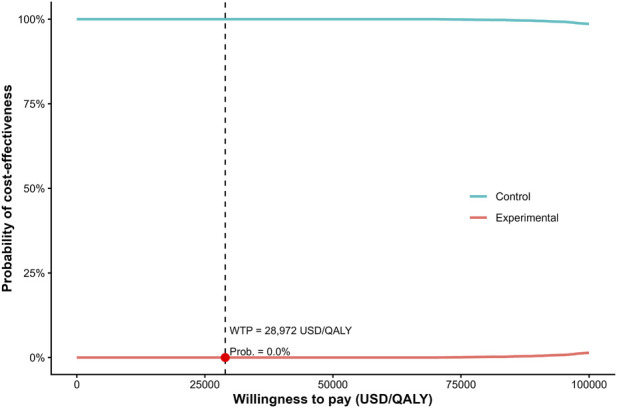
Cost-effectiveness acceptability curve.

The CEAC (Figure 4) further showed that the probability of nivolumab plus ipilimumab being the preferred cost-effective strategy at the current WTP threshold was close to zero.

### 3.3 Scenario analyses

#### 3.3.1 Scenario analysis of survival distribution model selection

The analysis of alternative survival model combinations showed that, across all 625 scenarios evaluated, the ICER consistently exceeded the WTP threshold in all cases. ICER values ranged from USD 117,456.88 to USD 204,894.89 per QALY, with a median value of USD 142,902.06 per QALY (Table 3).

**TABLE 3:** Distribution of ICERs under alternative survival model specifications.

Number of scenarios	Valid scenarios	Minimum ICER (USD/QALY)	25th percentile ICER	Median ICER	Mean ICER	75th percentile ICER	Maximum ICER	Probability below WTP (%)
625	625	117,456.88	132,215.52	142,902.06	147,317.62	160,660.65	204,894.89	0

ICER, incremental cost-effectiveness ratio; QALY, quality-adjusted life year.

In the base-case analysis, the ICER was USD 164,771.18 per QALY, which fell within the overall distribution and was close to the upper quartile. Although different survival model specifications influenced the absolute ICER estimates to some extent, they did not change the overall cost-effectiveness conclusion. These findings indicate that the results were not sensitive to the choice of survival extrapolation approach.

**FIGURE 5:**
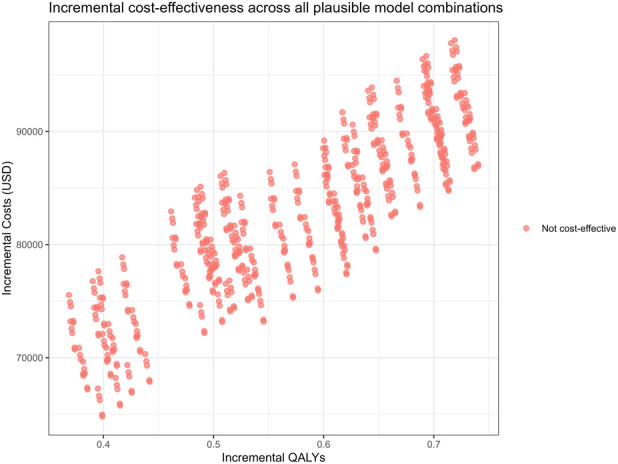
Cost-effectiveness plane of ICERs across survival model scenarios. Each point represents the incremental cost and incremental QALYs derived from a specific survival model combination. The dashed line indicates the WTP threshold of USD 28,972.38 per QALY.

**FIGURE 6:**
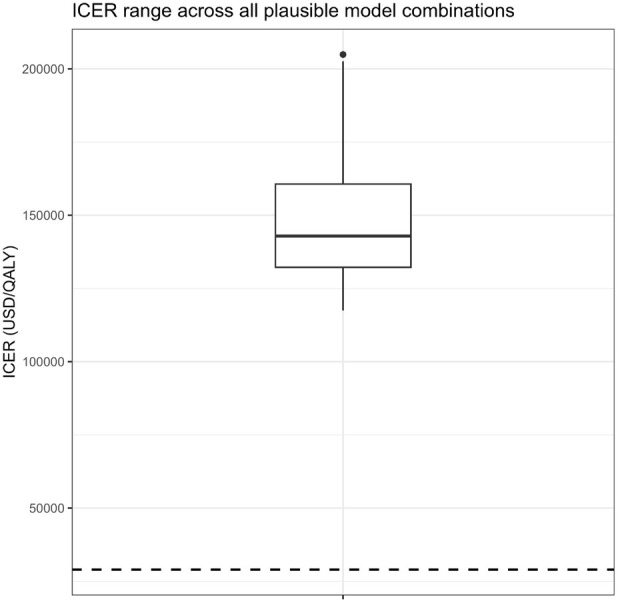
Distribution of ICERs across survival model scenarios. The boxplot presents the distribution of ICERs across all evaluated survival model combinations. The box represents the interquartile range (IQR), the horizontal line indicates the median, and whiskers denote the minimum and maximum values. The dashed line indicates the WTP threshold of USD 28,972.38 per QALY.

The distribution of ICERs across survival model combinations is illustrated in Figure 6, while the cost-effectiveness plane is presented in Figure 5.

Alternative time-horizon analyses showed that shortening the time horizon to 5 years resulted in an ICER of USD 272,503.87/QALY, whereas extending the time horizon to 15 years increased incremental QALYs to 0.55 and reduced the ICER to USD 144,806.80/QALY. However, under all time-horizon scenarios, the ICER remained substantially above the WTP threshold (Table 4).

**TABLE 4:** Scenario analyses under alternative time horizons.

Scenario	Time horizon	Incremental cost (USD)	Incremental QALYs	ICER (USD/QALY)
Base-case	10 years	80,446.82	0.49	164,177.18
Shorter horizon scenario	5 years	74,414.40	0.27	272,503.87
Extended horizon scenario	15 years	79,199.00	0.55	144,806.80

Abbreviations: ICER, incremental cost-effectiveness ratio; QALY, quality-adjusted life year.

#### 3.3.2 Scenario analysis of utility values

Scenario analyses using alternative utility inputs from the literature showed that the ICER remained above the WTP threshold across all scenarios (Table 5). Utility estimates were derived from published pharmacoeconomic studies and health-related quality-of-life analyses in patients with HCC. A summary of candidate utility values identified from the literature is provided in Supplementary Table 2.

**TABLE 5:** Scenario analysis of ICERs under alternative utility inputs.

Scenario	Utility (PFS)	Utility (PD)	Data source	Population context	Incremental cost (USD)	Incremental QALY	ICER (USD/QALY)
Base case	0.76	0.68	Literature consensus	China	80,446.82	0.50	164,177.18
Scenario 1	0.75	0.68	Lian et al., 2024	United States of America	80,446.82	0.49	164,804.80
Scenario 2	0.83	0.71	Cai et al., 2020	China (utility derived from Japanese population)	80,446.82	0.54	148,265.76

Abbreviations: ICER, incremental cost-effectiveness ratio.

Although ICER estimates varied under different utility assumptions, the overall variation was limited, and none of the alternative utility scenarios resulted in an ICER below the WTP threshold. The base-case ICER was within the range observed across all scenarios, indicating that the model results were relatively robust to alternative utility inputs.

#### 3.3.3 Structural uncertainty related to treatment and healthcare costs

Scenario analyses using alternative treatment and healthcare cost assumptions showed that the ICER was influenced by downstream treatment strategies and treatment exposure assumptions.

Assuming BSC only after disease progression increased the ICER to USD 183,705.74/QALY. Varying post-progression treatment costs by ±50% resulted in ICERs ranging from USD 145,774.06/QALY to USD 171,061.85/QALY.

Increasing AE management costs to two-fold of base-case values resulted in an ICER of USD 158,308.83/QALY. Reducing experimental treatment exposure to 80% of base-case levels lowered the ICER to USD 118,802.22/QALY; however, the ICER remained above the WTP threshold.

Varying non-drug medical costs by ±25% resulted in relatively small changes in ICER estimates, ranging from USD 158,319.94/QALY to USD 158,515.97/QALY (Table 6). Overall, the ICER remained above the WTP threshold across all evaluated scenarios.

**TABLE 6:** Scenario analyses under alternative treatment and healthcare cost assumptions.

Scenario	Incremental cost (USD)	Incremental QALYs	ICER (USD/QALY)
Base-case analysis	80,446.82	0.49	164,177.18
BSC only after progression	79,426.94	0.4324	183,705.74
Post-progression treatment cost −50%	72,490.37	0.4973	145,774.06
Post-progression treatment cost +50%	85,050.18	0.4973	171,061.85
AE management cost ×2	78,721.28	0.4973	158,308.83
Experimental treatment exposure reduced to 80%	59,083.87	0.4973	118,802.22
Non-drug medical cost −25%	78,726.81	0.4973	158,319.94
Non-drug medical cost +25%	78,824.32	0.4973	158,515.97

Abbreviations:AE, adverse event; BSC, best supportive care; ICER, incremental cost-effectiveness ratio; QALY, quality-adjusted life year. All scenario analyses were conducted by varying one structural assumption at a time while holding all remaining model parameters constant.

### 3.4 Threshold and Price–WTP decision-space analyses

Threshold and price–WTP decision-space analyses were conducted for first-line treatment costs in the experimental group while all other model parameters remained unchanged. ICERs decreased as treatment costs in the experimental group were progressively reduced.

At the base-case WTP threshold of USD 28,972.38/QALY (2× GDP *per capita*), the experimental regimen required an approximate price reduction of 67.05% to reach the WTP threshold. Under alternative WTP thresholds, the required price reductions were estimated to be 74.35% at 1× GDP *per capita* and 59.75% at 3× GDP *per capita*.

**FIGURE 7:**
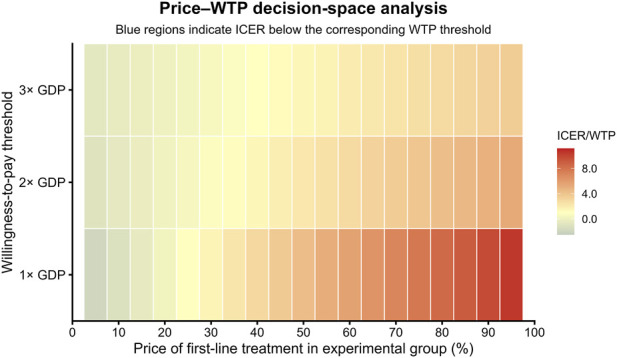
Price–WTP decision-space analysis for nivolumab plus ipilimumab The heatmap illustrates the relationship between first-line treatment price levels and WTP thresholds. Colors represent the ratio between the ICER and the corresponding WTP threshold (ICER/WTP). Lower ICER/WTP ratios indicate improved economic acceptability under the corresponding pricing scenario. WTP thresholds correspond to 1×, 2×, and 3× GDP *per capita* in China.

Additional price–WTP decision-space analyses were performed to evaluate the combined impact of treatment pricing and WTP thresholds on cost-effectiveness outcomes (Figure 7). Lower treatment prices and higher WTP thresholds were associated with lower ICERs. However, under most pricing scenarios, the ICER remained above commonly used Chinese WTP thresholds. Detailed threshold-analysis results under different WTP thresholds are presented in Table 7.

**TABLE 7:** Threshold price analysis under different WTP thresholds.

WTP threshold	WTP (USD/QALY)	Threshold price (%)	Required price reduction (%)	ICER at threshold (USD/QALY)
1× GDP	14,486.19	25.65	74.35	14,525.59
2× GDP	28,972.38	32.95	67.05	28,985.34
3× GDP	43,458.57	40.25	59.75	43,445.08

## 4 Discussion

### 4.1 Principal findings

In this study, a partitioned survival model (PSM) was developed to evaluate the cost-effectiveness of nivolumab plus ipilimumab versus TKI-based therapy as first-line treatment for unresectable HCC in China. The base-case analysis showed that nivolumab plus ipilimumab increased QALYs but resulted in substantially higher costs, with an ICER exceeding the conventional Chinese WTP threshold.

The unfavorable economic outcome was mainly driven by the high acquisition cost of dual immunotherapy relative to the incremental survival benefit. Post-progression treatment costs also contributed to the overall economic burden.

Across multiple structural uncertainty analyses, including 625 survival model combinations, alternative time horizons, downstream treatment scenarios, AE management assumptions, treatment exposure scenarios, and healthcare cost variations, the ICER remained above conventional Chinese WTP thresholds (Shao et al., 2023; Briggs et al., 2012; Bullement et al., 2019).

### 4.2 Comparison with previous studies

The findings of the present study were generally consistent with previous economic evaluations of immunotherapy-based strategies for advanced HCC in China. Wang et al. reported that most first-line immunotherapy regimens for unresectable HCC were unlikely to be cost-effective under conventional Chinese WTP thresholds because of high treatment costs and limited incremental QALY gains (Wang et al., 2023). Similar conclusions have also been reported in recent Chinese pharmacoeconomic studies involving immune checkpoint inhibitors and anti-vascular endothelial growth factor (VEGF)-based regimens (Liu et al., 2023).

Compared with previous studies, the present analysis incorporated more extensive structural uncertainty analyses related to long-term survival extrapolation and downstream treatment assumptions (Shao et al., 2023; Briggs et al., 2012; Bullement et al., 2019). In addition to evaluating 625 survival model combinations, alternative scenarios involving 5-year and 15-year time horizons, treatment exposure, downstream treatment costs, and healthcare cost variations were explored. A price–WTP decision-space analysis was also performed to evaluate the relationship between treatment pricing and economic outcomes.

### 4.3 Interpretation of structural uncertainty

Structural uncertainty remains an important challenge in oncology economic evaluations, particularly for immune checkpoint inhibitors with potentially durable survival benefits (Shao et al., 2023; Briggs et al., 2012; Bullement et al., 2019). Because follow-up durations in immunotherapy trials are often limited, long-term survival extrapolation may substantially influence cost-effectiveness outcomes (Bullement et al., 2019).

To address this issue, the present study systematically evaluated alternative survival model specifications rather than relying on a single parametric distribution. Across all evaluated survival model combinations, the ICER remained above conventional Chinese WTP thresholds. Similarly, analyses using alternative 5-year and 15-year time horizons did not materially alter the overall economic interpretation (Shao et al., 2023).

Uncertainty related to downstream treatment assumptions was also explored. Post-progression treatment patterns in the CheckMate 9DW trial were heterogeneous and involved multiple systemic and non-systemic treatment approaches. Although alternative downstream treatment assumptions influenced ICER estimates to some extent, the overall conclusions were similar across evaluated scenarios.

Treatment exposure and AE management burden may also differ between clinical trials and routine practice. However, reducing treatment exposure and increasing AE management costs did not reduce the ICER below conventional Chinese WTP thresholds.

### 4.4 Clinical and policy implications

HCC remains a major public health burden in China, with substantial disease-related mortality and healthcare expenditures (Long et al., 2024). Although dual immunotherapy provides clinically meaningful survival benefits, current treatment costs may limit its economic feasibility within the Chinese healthcare system.

The price–WTP decision-space analysis showed that the cost-effectiveness of nivolumab plus ipilimumab remained highly dependent on treatment pricing and WTP thresholds. Substantial price reductions would likely be required for the regimen to approach cost-effectiveness under conventional Chinese WTP thresholds.

These findings may provide supplementary evidence for healthcare decision-making and future pricing negotiations for immunotherapy-based strategies in advanced HCC.

### 4.5 Limitations

Several limitations should be acknowledged. First, the model was primarily based on the CheckMate 9DW trial population, which mainly included patients with preserved liver function and good performance status. Therefore, the findings may not be fully generalizable to all patients with unresectable HCC in routine clinical practice.

Second, despite extensive structural uncertainty analyses, long-term survival extrapolation remains inherently uncertain in oncology economic evaluations (Shao et al., 2023; Briggs et al., 2012; Bullement et al., 2019). Although multiple clinically plausible survival models and alternative time horizons were evaluated, uncertainty regarding long-term immunotherapy outcomes cannot be completely eliminated.

Third, simplified representative assumptions were required for post-progression treatment because detailed patient-level treatment pathways were unavailable. Although multiple downstream treatment scenarios were explored, real-world treatment patterns may still differ from those modeled in the present study.

Fourth, some AE-related disutility estimates were derived from studies conducted in other cancer populations because HCC-specific utility data remain limited. In addition, cross-country differences in health preferences may influence utility estimates.

Finally, the current model did not fully capture real-world variability in treatment adherence, severe immune-related AEs, or regional reimbursement differences.

## 5 Conclusion

At current prices, nivolumab plus ipilimumab is unlikely to be cost-effective as a first-line treatment for unresectable HCC in China under conventional WTPs. This conclusion remained broadly consistent across extensive structural and parameter uncertainty analyses, including alternative survival extrapolation models, time horizons, post-progression treatment assumptions, and healthcare cost scenarios. Substantial treatment price reductions would likely be required to improve the cost-effectiveness of dual immunotherapy within the Chinese healthcare system. These findings may provide supplementary evidence for future pricing negotiations and healthcare resource allocation decisions in China (Wei et al., 2018).

## Data Availability

The data presented in the study are deposited in the Zenodo repository (https://zenodo.org/records/20718914), accession number DOI: 10.5281/zenodo.20718914. The repository contains the model input parameters, reconstructed Kaplan–Meier survival data, and R code used for the cost-effectiveness analysis.
