# Supplementary material for: Cost-effectiveness of nivolumab plus ipilimumab versus lenvatinib or sorafenib for unresectable hepatocellular carcinoma in China: a modeling study with price threshold analysis
Source: Front Pharmacol. 2026 Jun 30;17:1860312. doi: 10.3389/fphar.2026.1860312 (PMC13364844; doi:10.3389/fphar.2026.1860312)
Supplement: Supplementary file 1 [file Supplementaryfile1.docx]

**Supplemental Materials Content**

Supplementary Table 1. Summary of survival model fitting, extrapolation performance, and selection results

| **Dataset** | **Model** | **LnL** | **AIC** | **BIC** | **Parameters** | **RMSE** | **TailSurv** | **In01** | **Monotonic** | **Candidate** | **Base-case** |
| --- | --- | --- | --- | --- | --- | --- | --- | --- | --- | --- | --- |
|  |  |  |  |  | **(natural scale)** |  |  |  |  |  |  |
| PFS_TG | exp | -288.79 | 579.58 | 583.39 | rate = 0.75 | NA | NA | NA | NA | No | No |
| PFS_TG | weibull | -282.97 | 569.94 | 577.57 | shape = 0.84;  scale = 1.33 | NA | NA | NA | NA | No | No |
| PFS_TG | gamma | -285.95 | 575.9 | 583.53 | shape = 0.84;  rate = 0.60 | NA | NA | NA | NA | No | No |
| PFS_TG | lnorm | -260.7 | 525.39 | 533.02 | meanlog = -0.30;  sdlog = 0.33 | 0.51 | 0.031 | TRUE | TRUE | Yes | No |
| PFS_TG | gompertz | -271.46 | 546.91 | 554.54 | shape = 0.54;  rate = 1.18 | NA | NA | NA | NA | No | No |
| PFS_TG | llogis | -267.62 | 539.24 | 546.87 | shape = 1.18;  scale = 0.71 | 0.51 | 0.043 | TRUE | TRUE | Yes | No |
| PFS_TG | gengamma | -250.57 | 507.15 | 518.59 | mu = -1.01;  sigma = 0.28; Q = -1.14 | NA | NA | NA | NA | No | No |
| PFS_TG | FP1 | -127.52 | 259.03 | 266.66 | power = 1 | NA | NA | NA | NA | No | No |
| PFS_TG | FP2 | -110.28 | 226.56 | 238.01 | powers = -0.5, -0.5 | NA | NA | NA | NA | No | No |
| PFS_TG | RCS | -127.48 | 268.95 | 295.65 | k = 7 | NA | NA | NA | NA | No | No |
| PFS_TG | RP-hazard | -242.44 | 494.88 | 513.96 | scale = hazard;  k = 3 | 0.49 | 0.035 | TRUE | TRUE | Yes | Yes |
| PFS_TG | RP-odds | -242.95 | 495.9 | 514.97 | scale = odds;  k = 3 | 0.48 | 0.062 | TRUE | TRUE | Yes | No |
| PFS_TG | RP-normal | -246.2 | 502.41 | 521.48 | scale = normal; | 0.49 | 0.047 | TRUE | TRUE | Yes | No |
|  |  |  |  |  | k = 3 |  |  |  |  |  |  |
| PFS_TG | GAM | -127.48 | 268.95 | 295.65 | k = 7 | NA | NA | NA | NA | No | No |
| PFS_TG | mix-cure | -242.44 | 494.88 | 513.96 | fallback = RP-hazard | NA | NA | NA | NA | No | No |
|  |  |  |  |  |  |  |  |  |  |  |  |
| PFS_CG | exp | -233.56 | 469.12 | 472.93 | rate = 0.96 | NA | NA | NA | NA | No | No |
| PFS_CG | weibull | -226.74 | 457.47 | 465.09 | shape = 1.22;  scale = 1.04 | 0.64 | 0 | TRUE | TRUE | Yes | No |
| PFS_CG | gamma | -225.53 | 455.07 | 462.68 | shape = 1.39;  rate = 1.42 | 0.64 | 0 | TRUE | TRUE | Yes | No |
| PFS_CG | lnorm | -227.01 | 458.02 | 465.64 | meanlog = -0.38;  sdlog = 0.05 | NA | NA | NA | NA | No | No |
| PFS_CG | gompertz | -230.81 | 465.62 | 473.23 | shape = 1.31;  rate = 0.80 | NA | NA | NA | NA | No | No |
| PFS_CG | llogis | -229 | 462 | 469.61 | shape = 1.64;  scale = 0.71 | NA | NA | NA | NA | No | No |
| PFS_CG | gengamma | -224.33 | 454.66 | 466.08 | mu = -0.16;  sigma = -0.06;  Q = 0.51 | 0.64 | 0 | TRUE | TRUE | Yes | No |
| PFS_CG | FP1 | -136.73 | 277.46 | 285.08 | power = -2 | NA | NA | NA | NA | No | No |
| PFS_CG | FP2 | -136.39 | 278.79 | 290.21 | powers = -2, 1 | NA | NA | NA | NA | No | No |
| PFS_CG | RCS | -143.5 | 301.01 | 327.66 | k = 7 | NA | NA | NA | NA | No | No |
| PFS_CG | RP-hazard | -217.69 | 445.39 | 464.43 | scale = hazard; | NA | NA | NA | NA | No | No |
|  |  |  |  |  | k = 3 |  |  |  |  |  |  |
| PFS_CG | RP-odds | -217.11 | 444.21 | 463.25 | scale = odds; | 0.64 | 0.002 | TRUE | TRUE | Yes | Yes |
|  |  |  |  |  | k = 3 |  |  |  |  |  |  |
| PFS_CG | RP-normal | -217.56 | 445.12 | 464.16 | scale = normal; | 0.64 | 0 | TRUE | TRUE | Yes | No |
|  |  |  |  |  | k = 3 |  |  |  |  |  |  |
| PFS_CG | GAM | -143.5 | 301.01 | 327.66 | k = 7 | NA | NA | NA | NA | No | No |
| PFS_CG | mix-cure | -217.69 | 445.39 | 464.43 | fallback = RP-hazard | NA | NA | NA | NA | No | No |
|  |  |  |  |  |  |  |  |  |  |  |  |
| OS_TG | exp | -399.83 | 801.67 | 805.48 | rate = 0.34 | NA | NA | NA | NA | No | No |
| OS_TG | weibull | -399.1 | 802.2 | 809.83 | shape = 0.93;  scale = 3.06 | 0.57 | 0.051 | TRUE | TRUE | Yes | No |
| OS_TG | gamma | -399.38 | 802.75 | 810.38 | shape = 0.92;  rate = 0.30 | 0.58 | 0.044 | TRUE | TRUE | Yes | Yes |
| OS_TG | lnorm | -399.01 | 802.02 | 809.64 | meanlog = 0.69;  sdlog = 0.45 | NA | NA | NA | NA | No | No |
| OS_TG | gompertz | -398.05 | 800.1 | 807.73 | shape = 0.85; | NA | NA | NA | NA | No | No |
|  |  |  |  |  | rate = 0.40 |  |  |  |  |  |  |
| OS_TG | llogis | -397.5 | 799.01 | 806.64 | shape = 1.13; | 0.53 | 0.14 | TRUE | TRUE | Yes | No |
|  |  |  |  |  | scale = 1.98 |  |  |  |  |  |  |
| OS_TG | gengamma | -397.21 | 800.42 | 811.87 | mu = 0.91;  sigma = 0.30; | 0.54 | 0.1 | TRUE | TRUE | Yes | No |
|  |  |  |  |  | Q = 0.46 |  |  |  |  |  |  |
| OS_TG | FP1 | -116.24 | 236.47 | 244.1 | power = 1 | NA | NA | NA | NA | No | No |
| OS_TG | FP2 | -112.95 | 231.89 | 243.33 | powers = -0.5, -0.5 | NA | NA | NA | NA | No | No |
| OS_TG | RCS | -116.24 | 246.47 | 273.17 | k = 7 | NA | NA | NA | NA | No | No |
| OS_TG | RP-hazard | -393.79 | 801.59 | 828.28 | scale = hazard; | NA | NA | NA | NA | No | No |
|  |  |  |  |  | k = 5 |  |  |  |  |  |  |
| OS_TG | RP-odds | -393.75 | 801.5 | 828.2 | scale = odds;  k = 5 | NA | NA | NA | NA | No | No |
| OS_TG | RP-normal | -393.51 | 801.03 | 827.73 | scale = normal; | 0.53 | 0.12 | TRUE | TRUE | Yes | No |
|  |  |  |  |  | k = 5 |  |  |  |  |  |  |
| OS_TG | GAM | -111.38 | 246.77 | 292.54 | k = 12 | NA | NA | NA | NA | No | No |
| OS_TG | mix-cure | -393.79 | 801.59 | 828.28 | fallback = RP-hazard | NA | NA | NA | NA | No | No |
|  |  |  |  |  |  |  |  |  |  |  |  |
| OS_CG | exp | -417.99 | 837.99 | 841.8 | rate = 0.42 | NA | NA | NA | NA | No | No |
| OS_CG | weibull | -410.22 | 824.43 | 832.05 | shape = 1.26;  scale = 2.29 | 0.71 | 0.002 | TRUE | TRUE | Yes | Yes |
| OS_CG | gamma | -410.41 | 824.82 | 832.44 | shape = 1.40;  rate = 0.64 | 0.71 | 0.004 | TRUE | TRUE | Yes | No |
| OS_CG | lnorm | -425.78 | 855.57 | 863.18 | meanlog = 0.47;  sdlog = 0.16 | NA | NA | NA | NA | No | No |
| OS_CG | gompertz | -412.63 | 829.25 | 836.87 | shape = 1.29;  rate = 0.31 | NA | NA | NA | NA | No | No |

Candidate models were required to demonstrate acceptable statistical fit, clinically plausible extrapolation behavior, absence of implausible OS/PFS crossing, and stable hazard trajectories over time. Base-case models were selected based on overall performance across statistical fit, visual inspection, and clinical plausibility.

Abbreviations: LnL, log-likelihood; AIC, Akaike information criterion; BIC, Bayesian information criterion; RMSE, root mean square error. TailSurv represents the extrapolated survival probability at the end of the 10-year base-case time horizon. NA indicates that the metric is not applicable or not estimable for the model. Parameters are reported on the model estimation scale. For certain flexible parametric models (e.g., generalized gamma), parameters may take negative values due to reparameterization and do not imply invalid or non-physical model behavior.

RMSE and extrapolation diagnostics were only calculated for candidate models with acceptable convergence and visual fit; therefore, some models are reported as NA.


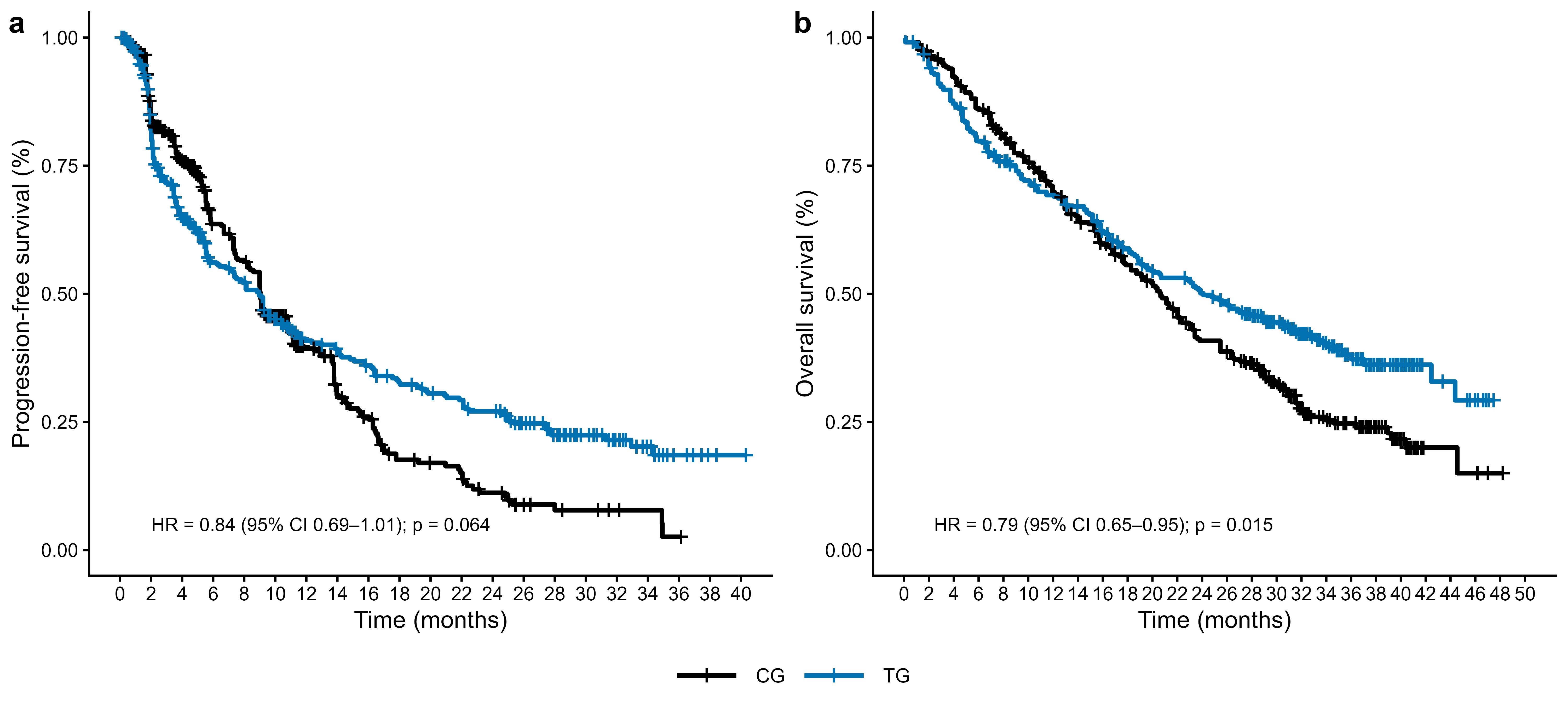


Supplementary Figure 1. Kaplan–Meier survival curves for progression-free survival (PFS) and overall survival (OS) in the phase III CheckMate 9DW trial.


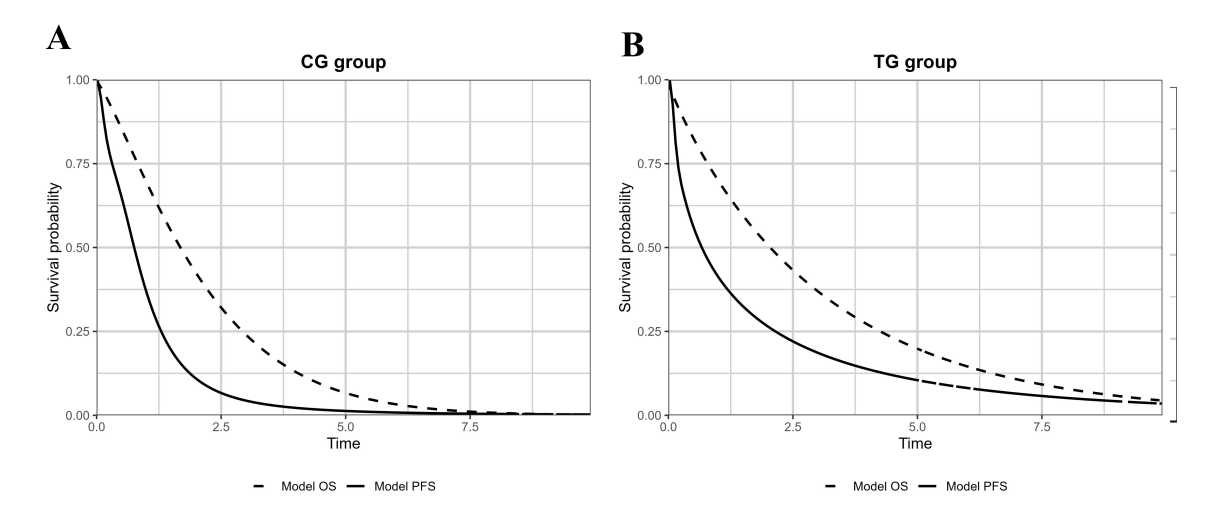


Supplementary Figure 2. Extrapolated progression-free survival and overall survival in the control and treatment groups.

Panel A shows the control group (CG), and Panel B shows the treatment group (TG). Dashed lines represent overall survival (OS), and solid lines represent progression-free survival (PFS). The selected models were RP-odds for PFS and Weibull for OS in the CG, and RP-hazard for PFS and gamma for OS in the TG. In both groups, the OS curve remained above the PFS curve throughout the extrapolation period, with no OS/PFS crossing observed during extrapolation.


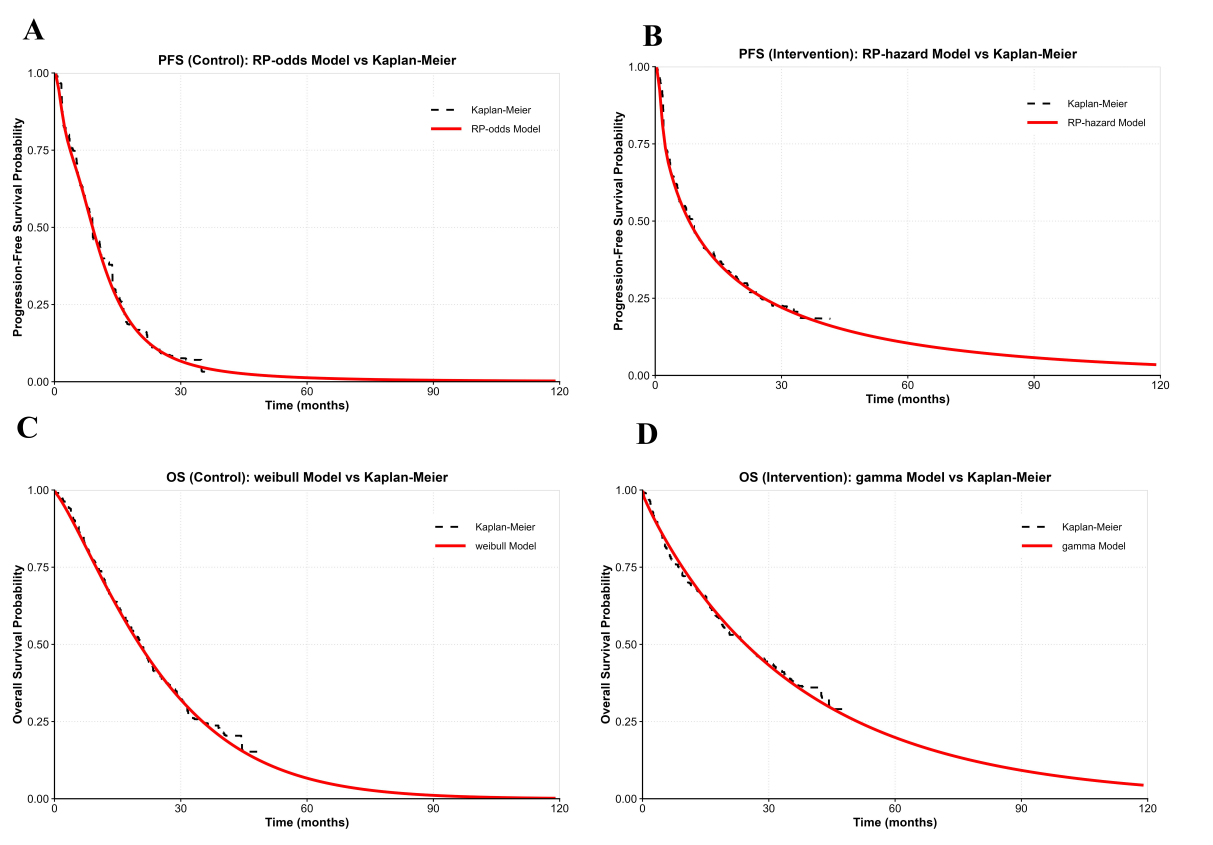


Supplementary Figure 3. Fitted and extrapolated survival curves.

Panels A–D show PFS and OS curves for the control and treatment groups. Dashed lines represent reconstructed Kaplan–Meier data, and solid lines represent model-based fitted and extrapolated survival curves. The selected models were RP-odds and RP-hazard for PFS, and Weibull and gamma for OS in the control and treatment groups, respectively.

Published studies reporting utility values for unresectable HCC were identified through targeted PubMed searches and review of relevant HTA reports.

Supplementary Table 2. Published health-state utility values used in scenario analyses for unresectable hepatocellular carcinoma

| **Study** | **Study type** | **Study / model setting** | **Utility for PFS** | **Utility for PD** | **Source** |
| --- | --- | --- | --- | --- | --- |
| Lian 2024 | Cost-effectiveness analysis | First-line systemic therapy for unresectable HCC | 0.75 | 0.68 | Lian et al. |
| Li 2022 | Cost-effectiveness analysis | Atezolizumab + bevacizumab versus nivolumab for advanced HCC | 0.76 | 0.68 | Li et al. |
| Wen 2024 | Cost-effectiveness analysis | First-line immunotherapy strategies for unresectable HCC | 0.76 | 0.68 | Wen et al. |
| Liu 2023 | Network meta-analysis and cost-effectiveness analysis | First-line systemic treatment strategies for unresectable HCC | 0.76 | 0.68 | Liu et al. |
| Liao 2024 | Cost-effectiveness analysis | Durvalumab + tremelimumab versus sorafenib for unresectable HCC | 0.76 | 0.68 | Liao et al. |
| Xu 2023 | Cost-effectiveness analysis | Sintilimab + IBI305 versus sorafenib for unresectable HCC | 0.76 | 0.68 | Xu et al. |
| Cai 2020 | Cost-effectiveness analysis | Lenvatinib versus sorafenib for advanced HCC | 0.83 | 0.71 | Cai et al. |
| Chen 2025 | Cost-effectiveness analysis | Anlotinib plus penpulimab versus sorafenib for unresectable HCC | 0.76 | 0.68 | Chen et al. |
| Liu 2025 | Cost-effectiveness analysis | Atezolizumab + bevacizumab versus sorafenib for advanced HCC | 0.84 | 0.71 | Liu et al. |
| Gaugain 2023 | Cost-utility analysis | Economic evaluation based on IMbrave150 population | 0.89 | 0.86 | Gaugain et al. |
| Qin 2018 | Cost-effectiveness analysis | Systemic chemotherapy versus sorafenib for advanced HCC | 0.76 | 0.68 | Qin et al. |

Supplementary Table S3. Drug acquisition cost assumptions and treatment schedules used in the economic model

| **Treatment setting** | **Drug/regimen** | **Dose and administration** | **Body weight assumption** | **Per-cycle cost assumption used in model (USD)** | **Weighted cost entering model**  **(USD)** |
| --- | --- | --- | --- | --- | --- |
| First-line experimental treatment | Nivolumab induction phase | 1 mg/kg Q3W | 67.7 kg | 910.21 | 910.21 |
| First-line experimental treatment | Ipilimumab induction phase | 3 mg/kg Q3W | 67.7 kg | 10,627.33 | 10,627.33 |
| First-line experimental treatment | Nivolumab maintenance phase | 480 mg Q4W | — | 4,840.11 | 4,840.11 |
| First-line control treatment | Lenvatinib | <60 kg: 8 mg QD; ≥60 kg: 12 mg QD | 67.7 kg | 470.32 | 399.77 |
|  |  | Receiving proportion: 85% |  |  |  |
| First-line control treatment | Sorafenib | 400 mg BID | — | 96.20 | 14.43 |
|  |  | Receiving proportion: 15% |  |  |  |
| Post-progression systemic therapy | Atezolizumab | 1200 mg Q3W | — | 4,767.44 | 1,811.63 |
|  |  | Receiving proportion: 38% of experimental arm |  |  |  |
| Post-progression systemic therapy | Bevacizumab | 15 mg/kg Q3W | 67.7 kg | 1,472.92 | 559.71 |
|  |  | Receiving proportion: 38% of experimental arm |  |  |  |
| Post-progression systemic therapy | Atezolizumab | 1200 mg Q3W | — | 4,767.44 | 2,479.07 |
|  |  | Receiving proportion: 52% of control arm |  |  |  |
| Post-progression systemic therapy | Bevacizumab | 15 mg/kg Q3W | 67.7 kg | 1,472.92 | 765.92 |
|  |  | Receiving proportion: 52% of control arm |  |  |  |

Abbreviations: BID, twice daily; QD, once daily; Q3W, every 3 weeks; Q4W, every 4 weeks.

Notes:

1. Because the CheckMate 9DW trial did not report detailed body weight distribution, body weight assumptions for lenvatinib dosing were informed by the REFLECT phase III trial, in which patients weighing ≥60 kg accounted for approximately 69%. This distribution was used to calculate the weighted average daily dose of lenvatinib.

2. Drug acquisition costs were estimated according to dosing schedules, assumed body weight, negotiated national prices, and a 21-day model cycle length.2 “Per-cycle cost assumption used in model” represents the estimated treatment cost before applying treatment proportions.

3. “Weighted cost entering model” represents the effective treatment cost incorporated into the model after weighting by the proportion of patients receiving each treatment regimen.

4. The proportions receiving post-progression systemic therapy were derived from the CheckMate 9DW trial.

5. Atezolizumab plus bevacizumab was modeled as representative post-progression systemic therapy because post-progression treatment patterns in CheckMate 9DW were heterogeneous and involved multiple treatment approaches.

6. Drug acquisition costs were calculated based on theoretical dosing requirements, and drug wastage or vial sharing was not considered in the base-case analysis.

Supplementary Table 4. CHEERS 2022 Reporting Checklist

| **Item No.** | **Section** | **Reporting Item** | **Location in Manuscript** |
| --- | --- | --- | --- |
| 1 | Title | Identification of the study as an economic evaluation | Title |
| 2 | Abstract | Structured summary including population, intervention, comparator, model, and results | Abstract |
| 3 | Background & objectives | Context, rationale, and study objective | Introduction |
| 4 | Health economic analysis plan | Model structure, survival extrapolation framework, and analytical assumptions | Methods 2.3–2.4 |
| 5 | Study population | Eligibility criteria and baseline characteristics | Methods 2.1 |
| 6 | Interventions & comparators | Treatment regimens, dosing, and treatment pathways | Methods 2.2 |
| 7 | Perspective | Analytic perspective (Chinese healthcare system) | Methods 2.5 |
| 8 | Time horizon | Base-case and alternative time horizons | Methods 2.3; Methods 2.7.1 |
| 9 | Discount rate | Discount rate applied to costs and outcomes | Methods 2.3 |
| 10 | Choice of health outcomes | QALYs and health-state utility estimation | Methods 2.5; Supplementary Table 2 |
| 11 | Measurement of effectiveness | Source of clinical effectiveness data | Methods 2.4 |
| 12 | Measurement and valuation of preference-based outcomes | Source and valuation of utility data (e.g., EQ-5D) | Methods 2.5; Methods 2.7.2; Supplementary Table 2 |
| 13 | Resource use & costs | Drug acquisition, monitoring, AE management, and supportive care costs | Methods 2.5; Table 1; Supplementary Table S3 |
| 14 | Currency & price year | Reporting of currency and price year (USD, 2025 price year) | Methods 2.5 |
| 15 | Model description | Partitioned survival model (PSM) | Methods 2.3 |
| 16 | Analytics & assumptions | Survival reconstruction, extrapolation methods, structural assumptions, and scenario analyses | Methods 2.4; Methods 2.7 |
| 17 | Characterising uncertainty | One-way sensitivity analysis, probabilistic sensitivity analysis, and structural uncertainty scenario analyses | Methods 2.6–2.8 |
| 18 | Heterogeneity | Characterisation of heterogeneity | Not applicable; no subgroup heterogeneity analyses were conducted |
| 19 | Results – Base case | ICER, total costs, and QALYs | Results 3.1 |
| 20 | Results – Uncertainty | Sensitivity and scenario analysis results | Results 3.2–3.4 |
| 21 | Results – Characterising heterogeneity | Results of subgroup heterogeneity analyses | Not applicable |
| 22 | Discussion | Key findings, limitations, and generalizability | Discussion |
| 23 | Funding & conflicts of interest | Funding sources and conflict of interest disclosures | Declarations |

**References**

1. Lian D, Gan Y, Xiao D, Xuan D, Liu S, Wei Y. Cost-effectiveness of first-line systemic therapies for unresectable hepatocellular carcinoma. Br J Clin Pharmacol. 2024 Dec 18. doi: 10.1111/bcp.16367.
2. Li Y, Liang X, Li H, Chen X. Atezolizumab plus bevacizumab versus nivolumab as first-line treatment for advanced or unresectable hepatocellular carcinoma: A cost-effectiveness analysis. Cancer. 2022 Nov 15;128(22):3995-4003. doi: 10.1002/cncr.34457.
3. Wen F, Huang P, Wu Q, Yang Y, Zhou K, Zhang M, Li Q. Promising first-line immuno-combination therapies for unresectable hepatocellular carcinoma: A cost-effectiveness analysis. Cancer Med. 2024 Aug;13(16):e70094. doi: 10.1002/cam4.70094.
4. Liu K, Zhu Y, Zhu H. Immunotherapy or targeted therapy as the first-line strategies for unresectable hepatocellular carcinoma: A network meta-analysis and cost-effectiveness analysis. Front Immunol. 2023 Jan 11;13:1103055. doi: 10.3389/fimmu.2022.1103055. PMID: 36713376; PMCID: PMC9874298.
5. Xu Z, Ye ZM, Tang YK, Deng DF, Zhou Q, Fang M, Zhang YY, Li XP. Cost-effectiveness analysis of sintilimab plus IBI305 versus sorafenib for unresectable hepatic cell carcinoma in China. Cancer Med. 2023 Jul;12(14):14871-14880. doi: 10.1002/cam4.5724.

6. Cai H, Zhang L, Li N, Zheng B, Liu M. Lenvatinib versus sorafenib for unresectable hepatocellular carcinoma: a cost-effectiveness analysis. J Comp Eff Res. 2020 Jun;9(8):553-562. doi: 10.2217/cer-2020-0041. Epub 2020 May 18. PMID: 32419473.

7. Chen J, Dong Z, Shou B, Huang Z, Huang L, Sun G. A cost-effectiveness analysis of a new and more effective treatment for unresectable hepatocellular carcinoma-from a Chinese perspective. Ann Med. 2025 Dec;57(1):2598903. doi: 10.1080/07853890.2025.2598903.

8.Liu L, Wang L, Ding Y, Zhang Q, Shu Y. Cost-effectiveness of atezolizumab plus bevacizumab versus sorafenib as first-line therapy in unresectable hepatocellular carcinoma in the US and Chinese setting: a modelling comparison study. BMJ Open. 2025 Mar 6;15(3):e094804. doi: 10.1136/bmjopen-2024-094804.

9.Gaugain L, Cawston H, Dubois de Gennes C, Sanchez Alvares J, Nahon P, Mazaleyrat B, Le Dissez C. Cost-utility analysis of atezolizumab with bevacizumab in untreated unresectable or advanced hepatocellular carcinoma in France. PLoS One. 2023 Jan 18;18(1):e0280442. doi: 10.1371/journal.pone.0280442.

10. Wang H, Jin C, Fang L, Sun H, Cheng W, Hu S. Health economic evaluation of stereotactic body radiotherapy (SBRT) for hepatocellular carcinoma: a systematic review. Cost Eff Resour Alloc. 2020 Jan 10;18:1. doi: 10.1186/s12962-019-0198-z.

11. Qin S, Kruger E, Tan SC, Cheng S, Wang N, Liang J. Cost-effectiveness analysis of FOLFOX4 and sorafenib for the treatment of advanced hepatocellular carcinoma in China. Cost Eff Resour Alloc. 2018 Aug 4;16:29. doi: 10.1186/s12962-018-0112-0.
